# Physiological Alterations in Deletion Mutants of Two Insulin-Like Peptides Encoded in *Maruca vitrata* Using CRISPR/Cas9

**DOI:** 10.3389/fphys.2021.701616

**Published:** 2021-07-02

**Authors:** Md. Abdullah Al Baki, Jin Kyo Jung, Yonggyun Kim

**Affiliations:** ^1^Department of Plant Medicals, Andong National University, Andong, South Korea; ^2^Division of Crop Cultivation and Environment Research, Department of Central Area Crop Science, National Institute of Crop Science, Rural Development Administration, Suwon, South Korea

**Keywords:** insulin-like peptide, trehalose, growth, reproduction, CRISPR/Ca9

## Abstract

Most insect species encode multiple insulin-like peptides (ILPs) that exhibit functional overlaps in mediating physiological processes such as development and reproduction. Why do they need multiple ILPs? To address this question, we tested a hypothesis of the requirement of multiple ILPs by generating mutants lacking individual ILP genes using the CRISPR/Cas9 technology. Two ILPs (ILP1 and ILP2) in the legume pod borer, *Maruca vitrata*, mediate similar physiological processes such as hemolymph sugar level, larval development, and adult reproduction. Individual knock-out mutants (ΔILP1 and ΔILP2) were generated. They showed successful development from larvae to adults. However, they suffered from high hemolymph sugar levels by enhancing trehalose titers in the hemolymph. The hyperglycemic effect was more evident in ΔILP2 mutants than in ΔILP1 mutants. Both mutants showed increased expression of *trehalose-6-phosphate synthase* but suppressed expression of *trehalase*. These mutants also showed altered expression patterns of insulin signaling components. Expression levels of *insulin receptor* and *Akt* genes were upregulated, while those of *FOXO* and *Target of rapamycin* genes were downregulated in these mutants. These alterations of signal components resulted in significant retardation of immature development and reduced body sizes. ΔILP1 or ΔILP2 females exhibited poor oocyte development. Bromo-uridine incorporation was much reduced at the germarium of ovarioles of these mutants compared with wild females. Expression of the *vitellogenin* gene was also reduced in these mutants. Furthermore, males of these deletion mutants showed impaired reproductive activities when they mated with wild-type females. These results suggest that both ILPs are required for mediating larval development and adult reproduction in *M. vitrata*.

## Introduction

Insect growth depends on favorable temperatures with a supply of nutrients. Favorable environmental cues promote immature growth through internal endocrine signals, mostly via insulin-like peptides (ILPs) (Okamoto and Yamanaka, 2015). In adult insects, ILPs can also mediate reproductive processes such as oogenesis and spermatogenesis (Ueishi et al., 2009; Lenaerts et al., 2019).

Insulin-like peptides encode a characteristic molecular structure containing a signal peptide, an A-chain, a C-chain, and a B-chain. After removing the C-chain via post-translational modification, a mature heterodimeric peptide consisting of A-B chains linked through a disulfide bond is produced and secreted in response to nutrient signals (Nässel and Vanden Broeck, 2016). The fat body is the central tissue that connects nutrients to ILP secretion from median neurosecretory cells in *Drosophila* (Rullifson et al., 2002) via a fat body-derived leptin-like protein called Unpaired 2 or a small peptide called CCHamide-2 in response to high fat or a high sugar diet (Rajan and Perrimon, 2012; Sano et al., 2015).

In target cells, signal transduction pathways mediated by insect ILPs are highly conserved among metazoans (Nijhout and Callier, 2013). Insect ILPs can use a transmembrane receptor tyrosine kinase called insulin receptor (InR) to activate both Ras-MAPK (mitogen-activated protein kinase) and PI3K/PKB (phosphatidylinositol-3-kinase/protein kinase B) pathways (Badisco et al., 2013). Once ILP binds to InR, it triggers the downstream ILP/insulin-like growth factor signal (IIS), beginning with autophosphorylation of InR, which then phosphorylates insulin receptor substrate (INS). Next, the phosphorylated INS can recruit PI3K to the membrane, where it phosphorylates phosphatidylinositol-4,5-bisphosphate into phosphatidylinositol-3,4,5-trisphosphate (PIP3). Accumulated PIP3 levels can then recruit phosphoinositide-dependent kinase (PDK), activating serine-threonine protein kinase (Akt). Akt then phosphorylates Forkhead Box O (FOXO) to prevent its translocation into the nucleus while phosphorylating the tuberous sclerosis complex to upregulate protein translation. Finally, the amino acid-mediated TOR (target of rapamycin) pathway independently activates the downstream signal of IIS, which promotes larval growth and adult reproduction (Das and Arur, 2017).

The first ILP was a small prothoracicotropic hormone of *Bombyx mori* (silkworm) known to be bombyxin (Nagasawa et al., 1984). Since then, several ILPs have been identified in each insect species. Genome analysis has revealed that each insect species possesses multiple ILPs: four in *Tribolium castaneum* (red flour beetle), seven in *Anopheles gambiae* (African malaria mosquito), eight in *Drosophila melanogaster* (fruit fly), 10 in *Acyrthosiphon pisum* (pea aphid), and 39 in *B. mori* (silkworm) (Wu and Brown, 2006). These multiple ILPs can mediate specific physiological processes. For example, in *Drosophila*, ILP6 mediates insect growth while ILP7 and ILP8 mediate reproduction (Veenstra, 2020). However, there are functional overlaps among different ILPs. Currently, it is unclear why each insect has multiple ILP signals to mediate a specific physiological process.

The legume pod borer, *Maruca vitrata* (Lepidoptera: Crambidae), is an insect pest infesting leaves, flowers, and pods of leguminous crops in subtropical and tropical regions (Sharma, 1998). At least two ILPs (*Mv-ILP1* and *Mv-ILP2*) are known in this species. They play crucial roles in mediating larval development and adult reproduction (Al Baki et al., 2019a,b). Trehalose, a main hemolymph sugar, fluctuates in its titer depending on the feeding rhythm. Therefore, it is regulated by both ILPs in *M. vitrata* (Al Baki et al., 2018a). These two ILPs also mediate oocyte development by stimulating mitotic division of stem cells and promoting vitellogenin (Vg) synthesis in *M. vitrata* (Al Baki et al., 2019b). Moreover, these two ILPs are similarly expressed in most developmental stages and different tissues (Al Baki et al., 2020). Thus, the functional redundancy of these two ILPs has been questioned, raising the physiological significance of multiple ILPs in this species.

Thus, the objective of this study was to clarify multiple ILPs in *M. vitrata* exhibiting functional overlap using individual deletion mutants against different ILPs with the clustered regularly interspaced short palindromic repeats/CRISPR-associated protein 9 (CRISPR/Cas9) technology.

## Materials and Methods

### Insect Rearing

Larvae of *M. vitrata* were reared with an artificial diet (Jung et al., 2009) under the following laboratory conditions: temperature, 25 ± 1°C; photoperiod, 16:8 h (L:D), and relative humidity, 60 ± 10%. Under such conditions, larvae of *M. vitrata* underwent five instars (L1–L5). Adults of *M. vitrata* were fed with 10% sucrose. L5 larvae of *M. vitrata* aged by days after final larval molt from day 1 to day 5 (L5D1–L5D5). For oviposition, kitchen paper towels were provided in adult cages.

### CRISPR/Cas9—Preparation of Single-Stranded Guide RNA (sgRNA)

Genomic DNA structures of *Mv-ILP1* and *Mv-ILP2* were analyzed by sequencing its open reading frame using genomic DNA (gDNA). The resulting exon sequences of *Mv-ILP1* and *Mv-ILP2* were submitted to an online design tool^1^ to detect the best target site. We selected 23 bp sgRNA containing protospacer adjacent motif (PAM) in each of *Mv-ILP1* and *Mv-ILP2*. A customized sgRNA was generated using a Guide-it sgRNA *In Vitro* Transcription kit (Takara Korea Biomedical, Seoul, South Korea) following two template DNA PCR amplification steps and consecutive *in vitro* transcription. Briefly, PCR was performed with a customer-designed forward primer [58 nucleotides = additional four nucleotides + T7 sequence (17 nucleotides) + target sequence (20 nucleotides) + two Gs + annealing site (15 nucleotides)] and a company-prepared reverse primer. The sgRNA was synthesized *in vitro* using T7 RNA polymerase according to the instructions of the manufacturer. The final amount of sgRNA was quantified with a spectrophotometer (NanoDrop, Thermo Fisher Scientific, Wilmington, DE, United States) and purified using a spin column provided with the kit.

### CRISPR/Cas9—Microinjection of sgRNA and Cas9 Protein

After female adults of *M. vitrata* have laid eggs on kitchen paper towels for 1 h at the scotophase (3–4 AM), eggs were dried in air for 10 min in a desiccator at room temperature (RT) and fixed on a cover glass. Sharp pointed (< 20 μm diameter) glass capillaries (10 μL quartz, World Precision Instrument, Sarasota, FL, United States) were prepared with a Narishige magnetic glass microelectrode horizontal puller (model PN30, Tritech Research, Los Angeles, CA, United States) for injection. Collected eggs were injected with microcapillaries using a sutter CO_2_-based pico pump injector (PV830, World Precision Instrument) under a stereomicroscope (SZX-ILLK200, Olympus, Tokyo, Japan). Injection (5 nL/egg) was performed using a mixture containing Cas9 (500 ng/μL) and sgRNA (50 ng/μL) through a micropyle. All injections were accomplished within 30 min after egg collection, including drying time. Treated eggs were then incubated at RT for 4 h before they were transferred to a growing chamber (25°C) for hatching.

### gDNA Extraction and Identification of Mutagenesis

Genomic DNA was extracted from the hemolymph of newly hatched L5 larvae with Chelax (Bio-Rad, Hercules, CA, United States). To extract gDNA, 30 μL hemolymph was mixed with 500 μL of 20% Chelex and heated at 100°C for 10 min. After cooling on ice for 2 min, the suspension was centrifuged at 10,000 × *g* for 3 min. The resulting supernatant was used as a gDNA sample. PCR was performed using 80 ng of gDNA as a template to amplify the region containing the CRISPR target site. PCR product was then cloned into a TOPO-2.1 vector (Invitrogen, Carlsbad, CA, United States) and sequenced. Primers used for mutagenesis analysis are listed in Supplementary Table 1.

### RNA Extraction and cDNA Preparation

Each L5 larva was used for total RNA extraction using Trizol reagent (Invitrogen, Carlsbad, CA, United States) according to the instructions of the manufacturer. First, extracted RNA was resuspended in nuclease-free water and quantified using a spectrophotometer (NanoDrop, Thermo Scientific, Wilmington, DE, United States). Next, RNA (1 μg) was used for cDNA synthesis with RT PreMix (Intron Biotechnology, Seoul, South Korea) containing oligo dT primer according to the instructions of the manufacturer.

### RT-PCR and RT-qPCR

RT-PCR was performed using DNA Taq polymerase (GeneALL, Seoul, South Korea) under the following conditions: initial denaturation at 94°C for 5 min followed by 35 cycles of denaturing at 94°C for 30 s, annealing at 52–58°C (Supplementary Table 1) for 30 s, extension at 72°C for 1 min, and a final extension step at 72°C for 10 min with gene-specific primers (Supplementary Table 1). Each RT-PCR reaction mixture (25 μL) consisted of cDNA template, dNTP (each 2.5 mM), 10 pmol of each forward and reversed primer, and Taq polymerase (2.5 unit/μL). In addition, β-actin (Supplementary Table 1) was used as a reference gene because of its relatively stable expression in different tissues of *M. vitrata* (Chang and Ramasamy, 2014).

All gene expression levels in this study were determined using a real-time PCR machine (Step One Plus Real-Time PCR System, Applied Biosystem, Singapore) following the guideline of Bustin et al. (2009). RT-qPCR was performed in a 20 μL reaction volume containing 10 μL of Power SYBR Green PCR Master Mix (Life Technologies, Carlsbad, CA), 5 μL of cDNA template (50 ng), and each 1 μL of forward and reverse primers (Supplementary Table 1). RT-qPCR cycling began with heat treatment at 95°C for 10 min followed by 40 cycles of denaturation at 95°C for 1 min, annealing at 52–58°C (Supplementary Table 1) for 30 s, and extension at 72°C for 40 s. The expression level of β-actin as an internal control was used to normalize target gene expression levels under different treatments. PCR products were assessed by melting curve analysis. Quantitative analysis was performed using the comparative Ct (2^–ΔΔCt^) method (Livak and Schmittgen, 2001).

### Hemolymph Collection and Trehalose Measurement Using HPLC

Hemolymph was collected after cutting the first abdominal prolegs of mutant L5 larvae. A few phenylthiourea (Sigma-Aldrich Korea, Seoul, South Korea) granules were added to the hemolymph sample to prevent coagulation. After centrifugation at 400 × *g* for 3 min, the supernatant plasma was diluted 20× with distilled water. The diluted plasma sample was cleaned with a Sep-Pak C18 cartridge (Walters Associates, Milford, MA, United States). Trehalose was identified and quantified using high-performance liquid chromatography (HPLC) (BioLC, Dionex, Sunnyvale, CA, United States) with the main column (CarboPacMA1, 4 × 250 mm, Dionex) and a guard column (CarboPacMA1, 4 × 50 mm, Dionex) following the method described by Park and Kim (2013). The injection volume of the sample was 25 μL. NaOH (400 mM) was used as an elution buffer at a constant rate of.4 mL/min. Pulse amperometry mode of an electrochemical detector (ED40, Dionex) was used for detecting trehalose and other sugars.

### *In vitro* Organ Culture and Bromouridine (BrdU) Incorporation

For *in vitro* organ culture, 5-day-old virgin females were used. Ovaries were collected and cultured in a TC-100 insect cell culture medium (Hyclone, Daegu, South Korea) containing 10 μM BrdU (Sigma-Aldrich, Seoul, South Korea) for 24 h at 25°C. These ovaries were then fixed with 3.7% paraformaldehyde in a wet chamber under darkness at 25°C for 60 min. After washing with 100 mM phosphate-buffered saline (PBS, pH 7.4), ovarioles were permeabilized with.2% Triton X-100 in PBS at RT for 20 min. These ovaries were then rinsed with PBS three times, blocked with 5% skim milk (MB cell, Seoul, South Korea) in PBS at RT for 1 h, washed once with PBS, and incubated with mouse anti-BrdU antibody (BD Bioscience, San Jose, CA) diluted 1:15 in blocking solution for 1 h. Unbound anti-BrdU was washed off with PBS three times. Ovaries were then incubated with FITC-conjugated anti-mouse antibody (Sigma-Aldrich, Gangnam-Gu, South Korea) diluted 1:300 in blocking solution at RT for 1 h. After washing off the unbound anti-mouse antibody with PBS three times, stained ovarian cells were observed under a fluorescence microscope (DM2500, Leica, Wetzlar, Germany) at 200× magnification.

### Test for the Reproductive Activity of Mutant Males

After confirmation of deletion mutants during the L5 stage by gDNA sequencing, newly emerged mutant adults were separated and used to obtain inbred or hybrid lines by reciprocal matings with mutant or wild-type adults having the same age. Each cross used one male and two females. Total eggs laid by a single female and subsequent hatching rate were recorded. Each mating treatment was replicated three times.

### Statistical Analysis

All results are expressed as mean ± SD. They were plotted using Sigma Plot (Systat Software, San Jose, CA, United States). Means were compared by least square difference (LSD) test based on one-way analysis of variance using PROC GLM of SAS program (SAS Institute, 1989) and discriminated at Type I error = 0.05.

## Results

### Generation of CRISPR Mutants Against Two ILP Genes

**FIGURE 1:**
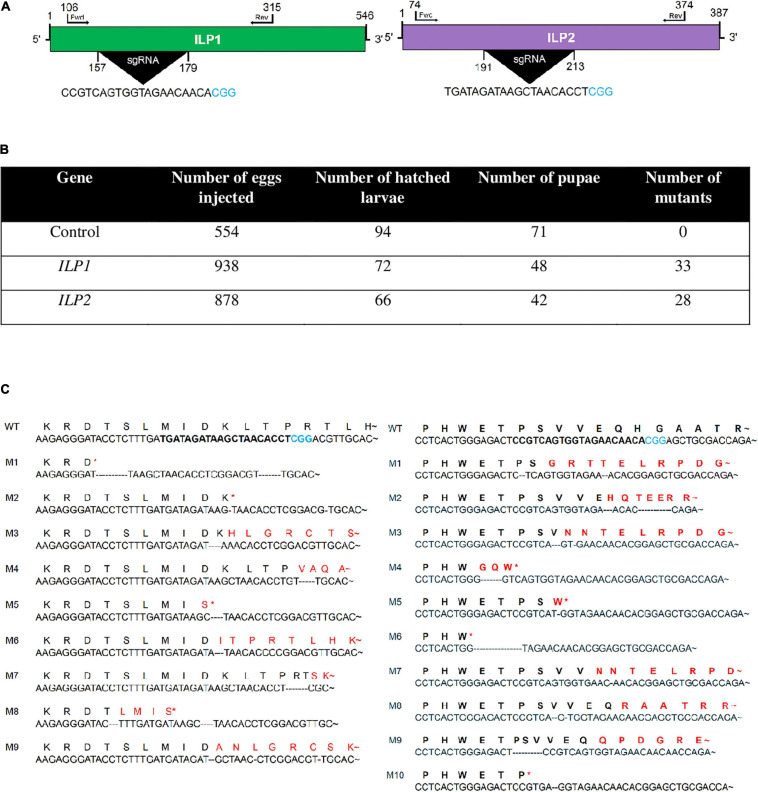
Mutagenesis of two insulin-like peptide genes (*Mv-ILP1* and *Mv-ILP2*) in *M. vitrata* by CRISPR-Cas9. **(A)** Schematic diagram of sgRNA targeting sites of *Mv-ILP1* and *Mv-ILP2*. These targeted sequences are highlighted in black. PAM sequence is shown in blue color. **(B)** Statistics of mutagenesis. **(C)** Mutation in target sites after CRISPR-Cas9. Fifth instar larvae were bled to collect hemocytes, from which genomic DNA was extracted for sequencing analysis. Ten mutants (“M1–M10”) for *Mv-ILP1* locus and nine mutants (“M1–M9”) for *Mv-ILP2* locus are compared with wild-type (“WT”) control in the DNA region. Deduced amino acid sequences in the target region are compared among WT and the two mutants. Asterisk indicates early termination. Trehalose peak is indicated with red-colored arrow.

To confirm the physiological functions of two ILPs in the development and reproduction of *M. vitrata*, CRISPR/Cas9 mutagenesis technology was applied to generate two mutants with deletion of either *Mv-ILP1 or Mv-ILP2* from its genome (Figure 1). To design single-stranded guide RNA (sgRNA), genome structures within their open reading frames (ORFs) were assessed by sequencing gDNA that showed no intron. sgRNA was designed in the size of 20-mer near the PAM motif (Figure 1A). Newly laid eggs (< 5 h) were injected with sgRNA and Cas9 protein through micro-capillaries in a 5 nL volume. For control eggs injected with the only sgRNA, the hatching rate was about 17.0% (Figure 1B). For eggs injected with sgRNA + Cas9 protein, the hatching rate was only 7.7% for *Mv-ILP1* or 7.5% for *Mv-ILP2*. All larvae developed into fifth instar larvae. Their genomes were assessed to determine the efficacy of CRISPR mutagenesis by sequencing the target region using forward (Fwd) and reverse (Rev) primers. CRISPR mutagenesis against *Mv-ILP1* and *Mv-ILP2* resulted in mutation rates of 45.8% and 42.4%, respectively. In addition, these mutants exhibited deletion mutation of nucleotide in the target region. Ten (M1–M10) and nine (M1–M9) different types of deletion mutants were generated in *Mv-ILP1* and *Mv-ILP2*, respectively (Figure 1C). Mutants (ΔILP1) in *Mv-ILP1* had deletions ranging from two nucleotides (M5) to 16 nucleotides (M6), in which four mutants (M4, M5, M6, and M10) had early termination signal by a subsequent frameshift mutation. Similarly, *Mv-ILP2* mutants (ΔILP2) had deletions ranging from two nucleotides (M2) to 17 nucleotides (M1), in which four mutants (M1, M2, M5, and M8) had early termination signal. All the deletion mutants were used to obtain next-generation mutants in each ILP gene for subsequent bioassays to monitor physiological alterations.

### Alteration of Hemolymph Trehalose Levels in Two ILP Deletion Mutants

**FIGURE 2:**
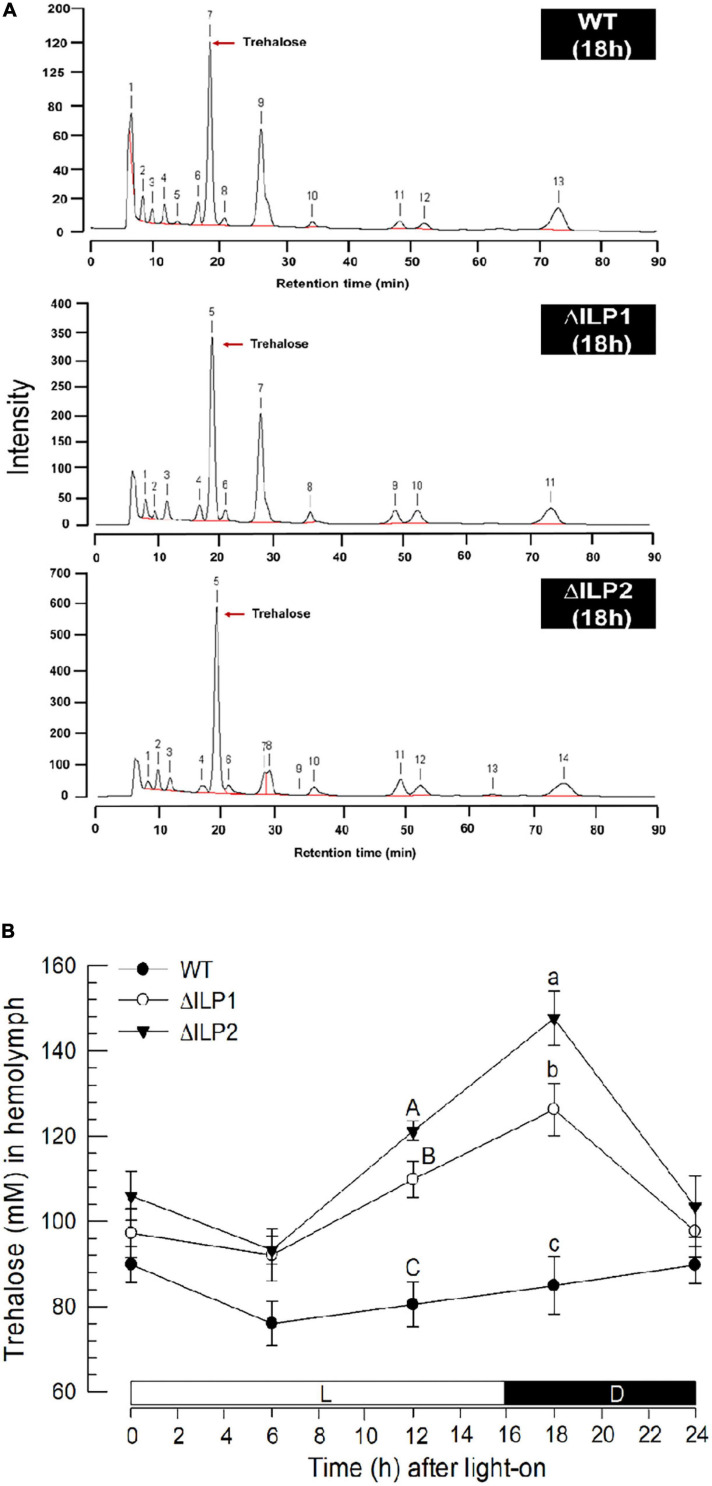
Influence of deletion mutation of two insulin-like peptides (Mv-ILP) genes on hemolymph trehalose titers in *M. vitrata* larvae. Hemolymph was collected by cutting the first abdominal proleg of mutant L5 larvae at 18 h after light-on (L:D = 16:8 h). **(A)** Representative chromatograms of hemolymph samples were collected from control and two mutants: *Mv-ILP1* (“ΔILP1”) and *Mv-ILP2* (“ΔILP2”). **(B)** Trehalose titers in hemolymph at different time points after dsRNA injection. All treatments were independently replicated three times. Different letters above standard deviation bars indicate significant differences among means at each time point with Type I error = 0.05 (LSD test). LSD, least square difference.

Trehalose was the major hemolymph sugar of wild or mutant larvae of *M. vitrata* when measured at 2 h after light-off (18 h after light-on, Figure 2A). Hemolymph trehalose levels were relatively constantly maintained at about 80 mM in control larvae, showing a slight fluctuation from 75 to 90 mM (Figure 2B). However, two ILP mutants had slightly higher trehalose levels during most photophase and increased significantly (*P* < 0.05) at around light-off. At 2 h after light-off, trehalose level was about 120 mM in ΔILP1 and about 150 mM in ΔILP2 while control larvae kept the basal level of trehalose titer in the hemolymph.

**FIGURE 3:**
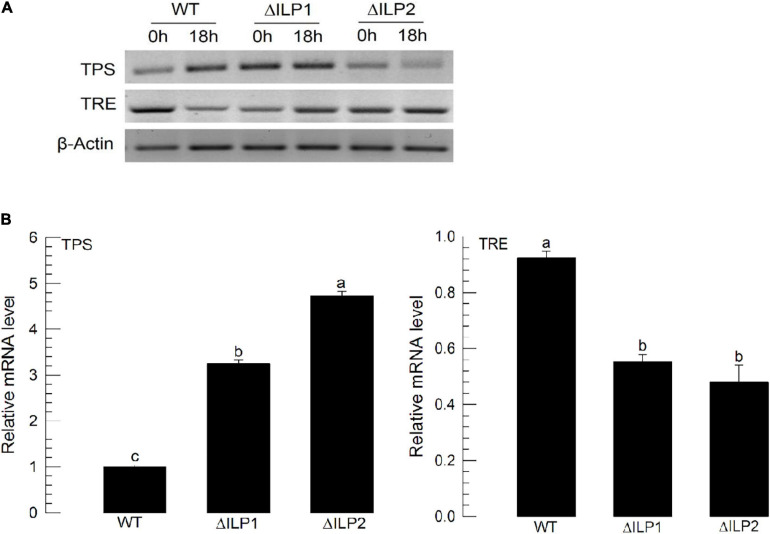
Influence of deletion mutation in two insulin-like peptides (Mv-ILP) genes on expression levels of trehalose metabolism-associated genes [trehalose-6-phosphate synthase (TPS) and trehalase (TRE)] in *M. vitrata* larvae. L5D1 mutant larvae of *Mv-ILP1* (“ΔILP1”) and *Mv-ILP2* (“ΔILP2”) were used for analysis. **(A)** *TPS* and *TRE* expression levels at 0 and 18 h after light-on in all treatments by RT-PCR. **(B)** Change of *TPS* and *TRE* expression levels at 18 h after light-on by real-time qPCR. β-actin gene was used as a reference gene to normalize target gene expression levels. All treatments were independently replicated three times. Different letters above standard deviation bars indicate significant differences among means at Type I error = 0.05 (LSD test). LSD, least square difference.

To explain the hypertrehalosemia in two deletion mutants, expression levels of two genes associated with trehalose synthesis (trehalose-6-phosphate synthase: TPS) and degradation (trehalase: TRE) were assessed (Figure 3). These two genes were expressed in both wild-type and mutant larvae (Figure 3A). When RT-qPCR quantified their expression levels, both mutants showed significantly (*P* < 0.05) increased *TPS* expression levels but downregulated *TRE* expression levels (Figure 3B). Especially, ΔILP2 mutants showed significantly more upregulation of *TSP* expression (*P* < 0.05) than ΔILP1 mutants.

### Dysregulated Gene Expression of IIS Components in Two ILP Deletion Mutants

**FIGURE 4:**
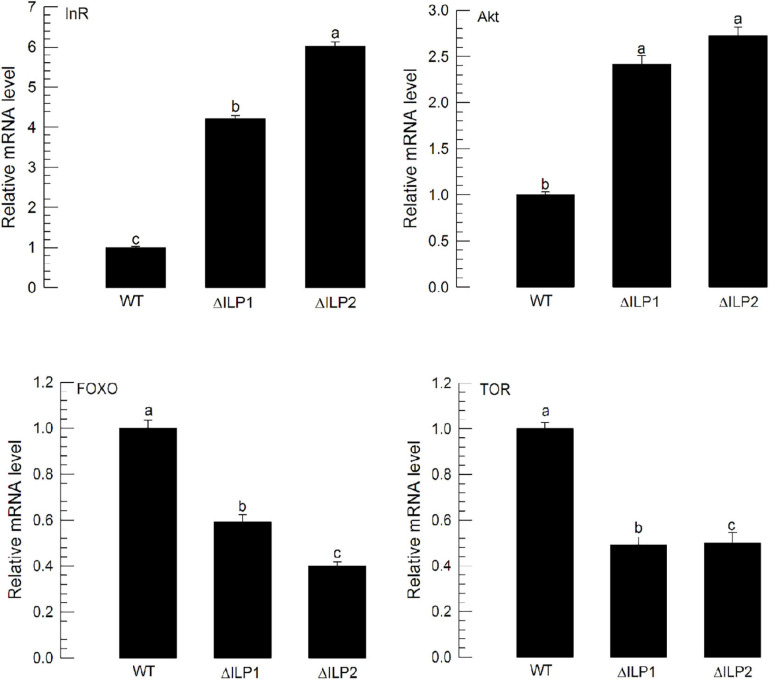
Influence of deletion mutation in two insulin-like peptides (Mv-ILP) genes on expression levels of four IIS component genes (*InR*, *Akt*, *FOXO*, and *TOR*) in *M. vitrata* larvae. These four gene expression levels in L5D1 larvae of mutants were quantified compared with those in the WT at 18 h after light-on. β-actin gene was used as a reference gene for RT-qPCR to validate cDNA integrity. Each treatment was replicated three times. Different letters above standard deviation bars indicate significant differences among means at Type I error = 0.05 (LSD test). LSD, least square difference.

The lack of ILP in ΔILP1 and ΔILP2 mutants might change the expression profile of ILP signaling components. To test this hypothesis, expression levels of four IIS component genes were assessed by RT-qPCR (Figure 4). Compared with control larvae, ΔILP1 and ΔILP2 mutants exhibited almost fourfold and sixfold increases of *Mv-InR* expression levels. In addition, expression levels of *Mv-Akt* were increased almost 2.5-fold in these mutants. However, gene expression levels of *Mv-FOXO* and *Mv-TOR* were significantly (*P* < 0.05) suppressed in these mutants. These supported the hypothesis of IIS dysregulation in ILP mutants.

### Impaired Immature Development and Adult Reproduction of Two ILP Deletion Mutants

**FIGURE 5:**
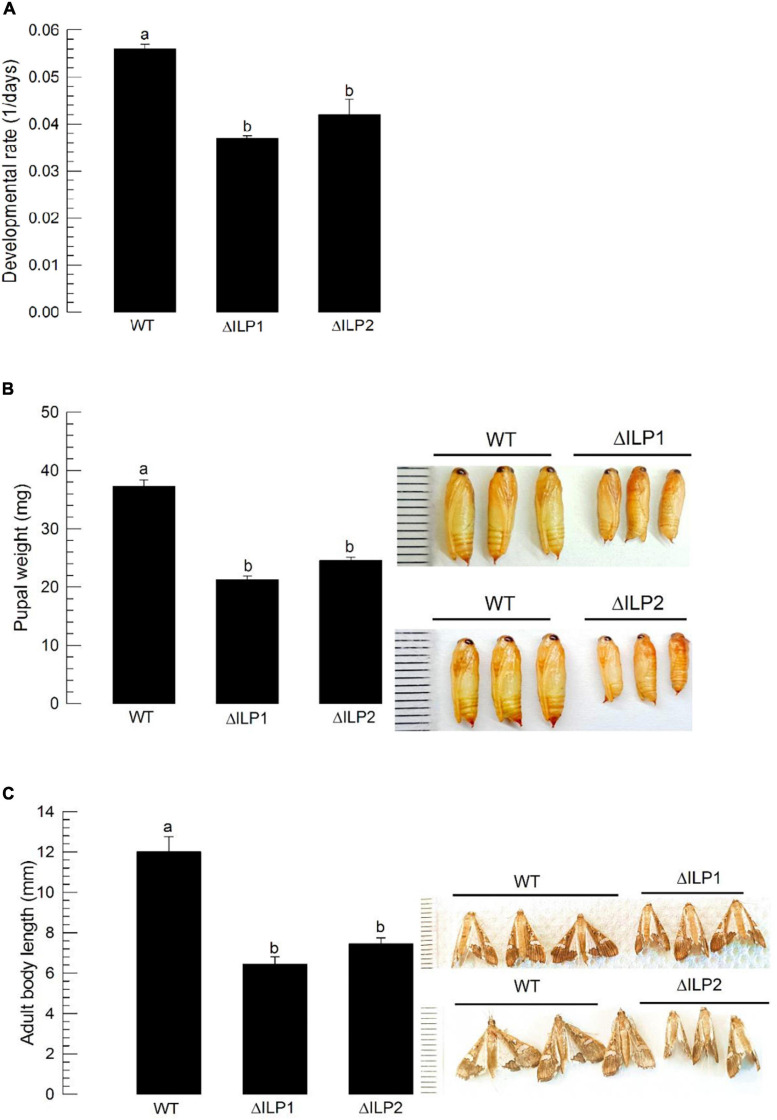
Developmental alteration for mutants with deletion of two insulin-like peptides (Mv-ILP) genes in *M. vitrata*: deletion of *Mv-ILP1* (“ΔILP1”) and deletion of *Mv-ILP2* (“ΔILP2”). **(A)** Retardation of larval development. The developmental rate was calculated by the inverse of the time period (days) from larval hatch to pupation. **(B)** Reduced pupal size (*n* = 10). Pupal weight was measured within 8 h after pupation. **(C)** Reduced adult female size at 5 days after adult emergence (*n* = 10). Different letters above standard deviation bars indicate significant differences among means at Type I error = 0.05 (LSD test). LSD, least square difference.

Dysregulation in IIS component expression might lead to altered immature development of *M. vitrata*. To test this hypothesis, larval periods were measured for three treatment groups (control, ΔILP1, and ΔILP2) to estimate developmental rate (Figure 5A). The two deletion mutants developed to pupae significantly (*P* < 0.05) slowly compared with the control. In addition, developed pupae of mutants were significantly (*P* < 0.05) smaller in size and weight than control pupae (Figure 5B). Adults of mutants were also significantly (*P* < 0.05) smaller than control adults (Figure 5C).

**FIGURE 6:**
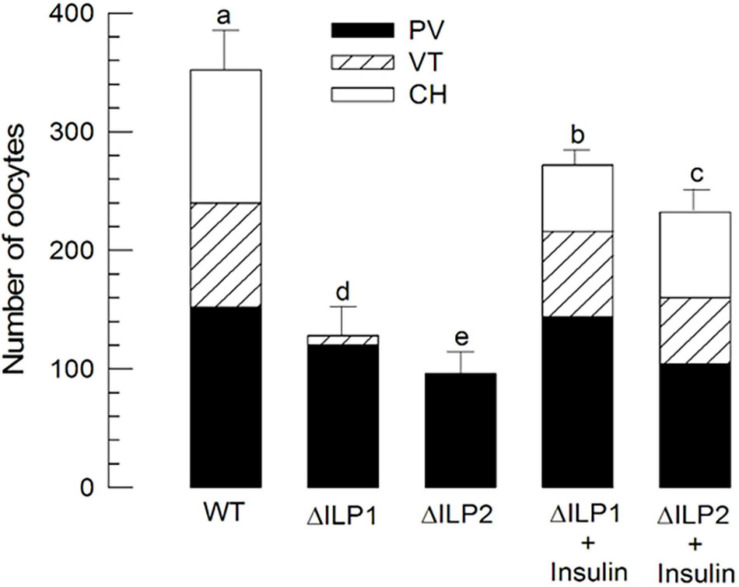
Alteration in oocyte development of *M. vitrata* mutants with deletion mutation for two insulin-like peptides (Mv-ILP) genes: *Mv-ILP1* (“ΔILP1”) and *Mv-ILP2* (“ΔILP2”). Quantitative analysis (*n* = 10) of oocyte development by counting the number of previtellogenic (“PV”), vitellogenic (“VT”), and chorionated (“CH”) oocytes. Total oocyte number was calculated by multiplying the oocyte number in each ovariole by eight because of the presence of eight ovarioles per pair of ovaries. Ovaries were collected from 5-day-old females of wild type (“WT”), mutant, or after the addition of porcine insulin. Insulin was injected to newly emerged (< 5 h after emergence) females with a microsyringe at 3 μg/individual. Different letters above standard deviation bars indicate significant differences among means at Type I error = 0.05 (LSD test). LSD, least square difference.

Oocyte development of *M. vitrata* underdoes three stages: previtellogenesis, vitellogenesis, and choriogenesis (Al Baki et al., 2019b). Compared with control (“WT”) females, both mutants (ΔILP1 and ΔILP2) showed poorly developed ovarioles in vitellogenic and chorionated oocytes (Figure 6). However, an injection of vertebrate insulin (3 μg/insect) significantly (*P* < 0.05) rescued the oocyte development.

**FIGURE 7:**
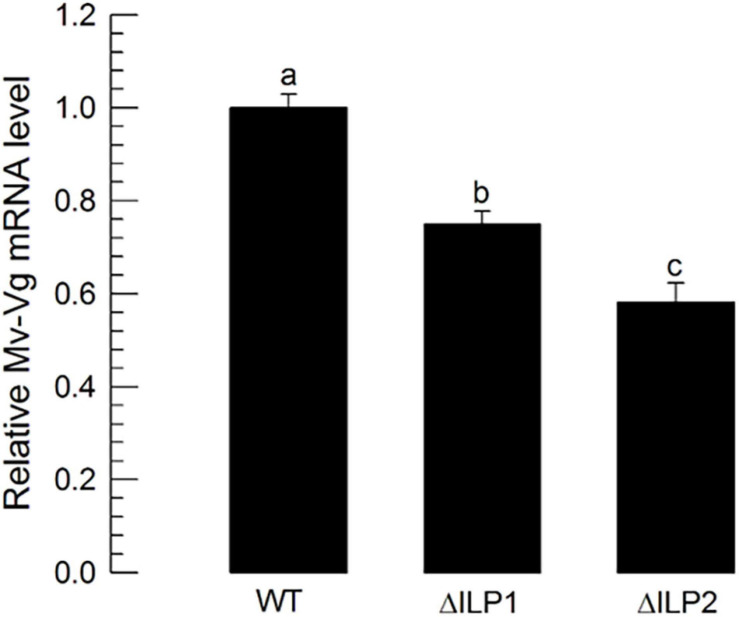
Influence of deletion mutation of two insulin-like peptides (Mv-ILP) genes on oocyte development at vitellogenic stages of *M. vitrata* females: mutants of *Mv-ILP1* (“ΔILP1”) and *Mv-ILP2* (“ΔILP2”). Reduction in vitellogenesis in mutants by measuring expression levels of vitellogenin (*Mv-Vg*). Real-time qPCR quantified expression levels of Vg in 5-day-old females. All treatments were independently replicated three times. β-actin was used as a reference gene in RT-qPCR to normalize the target gene expression level. Different letters above standard deviation bars indicate significant differences among means at Type I error = 0.05 (LSD test). LSD, least square difference.

To monitor the multiplication of oocytes at the germarium of ovarioles, DNA replication during cell division was stained through BrdU incorporation (Supplementary Figure 1). Control females had significantly higher numbers of BrdU-labeled cells with higher intensities than the two mutants. To analyze the difference in vitellogenesis, vitellogenin (*Mv-Vg*) expression in the abdomen of females was assessed by RT-qPCR (Figure 7). Both mutants showed significantly (*P* < 0.05) decreased *Mv-Vg* expression levels compared with control females.

### Influence of ILP Mutation on Male Reproduction

**FIGURE 8:**
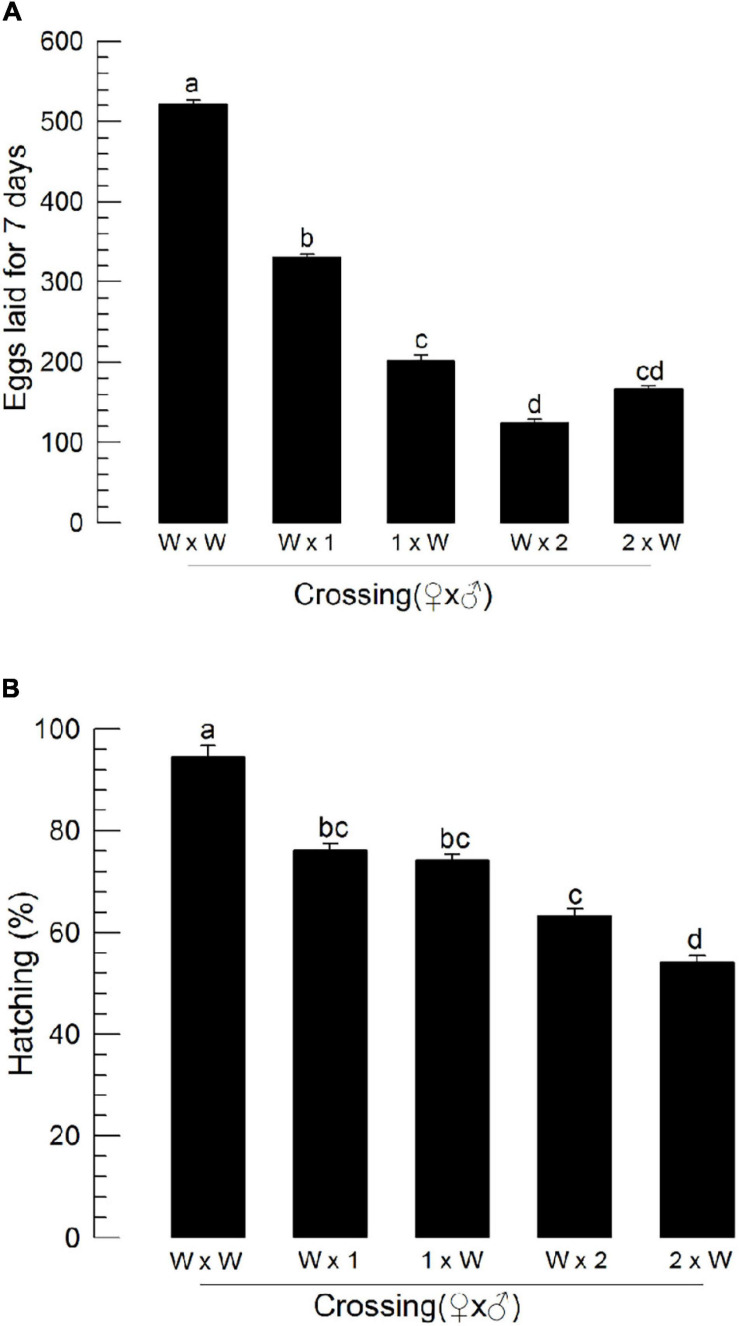
Influence of deletion mutation of two insulin-like peptide genes on the reproductive activity of *M. vitrata* males by reciprocal crosses with wild-type (“W”) females. “W × 1” and “W × 2” represent W females mating with mutants of *Mv-ILP1* and *Mv-ILP2*, respectively. The reproductive activity was assessed based on the number of laid eggs for 7 days **(A)** and subsequent egg hatching rate **(B)**. Newly emerged mutant adults were used for this experiment. One virgin male and two virgin females were used for each mating. All crosses were independently replicated three times. Different letters above standard deviation bars indicate significant differences among means at Type I error = 0.05 (LSD test). LSD, least square difference.

To assess male reproduction of CRISPR mutants, these mutants were then allowed to mate with wild-type individuals by reciprocal crosses (Figure 8). As expected, when mutant females mated with wild-type males, they exhibited poor reproductive activities. Interestingly, when wild-type females mated with ΔILP1 or ΔILP2 males, they (“W × 1” or “W × 2”) showed significant (*P* < 0.05) decreases for the number of laid eggs (Figure 8A) and subsequent hatch rate (Figure 8B).

## Discussion

Insects encode multiple ILPs that mediate various insect physiological processes (Wu and Brown, 2006). In *M. vitrata*, two ILPs have been identified (Al Baki et al., 2018a, 2020). They play important roles in mediating immature growth and adult reproduction (Al Baki et al., 2018a, 2020). Furthermore, their expression patterns are similar among different developmental stages and tissues (Al Baki et al., 2019a). Furthermore, IIS components responding to ILPs are well conserved in *M. vitrata* (Al Baki et al., 2020). Previous studies using the RNAi approach have shown that these two ILPs mediate similar physiological processes without discriminating their independent roles. Thus, we hypothesized that these two ILPs are required to perform their mediation roles in the physiological processes of *M. vitrata*. To clarify whether they might have independent roles during entire developmental stages, we constructed two mutant lines deleting each of these two ILPs using the CRISPR/Cas9 technique.

CRISPR/Cas9 generated ILP mutants showed deletions of several nucleotides in their ORFs, leading to altered amino acid sequences or early chain termination in the two *ILP* loci of *M. vitrata*. CRISPR/Cas9 mutagenesis uses a prokaryotic adaptive machinery that enables bacteria and archaeal species to resist invading viruses and phages or plasmids (Doudna and Charpentier, 2014). The CRISPR/Cas9 system is an invaluable system for precise gene editing of diverse species, including insects, because it requires less time and effort than other genome editing techniques such as zinc finger nucleases and transcription activator-like effector nucleases (Sun et al., 2017). The current study used non-homologous end joining (NHEJ) mutagenesis of CRISPR/Cas9, in which sgRNA designed based on *Mv-ILP1* or *Mv-ILP2* sequences could specifically bind to target locus. Cas9 then could cut a specific site of the target locus. The resulting cleaved ends were then joined in a manner of NHEJ by endogenous DNA ligase activity. This NHEJ resulted in the deletion of 2–16 nucleotides for *Mv-ILP1* or 2–17 nucleotides for *Mv-ILP2*, leading to missense or nonsense mutations.

These two deletion mutants lost hemolymph sugar levels of *M. vitrata* and led to a hypertrehalosemic state. A disaccharide, trehalose, is the main hemolymph sugar of *M. vitrata* larvae, in which its titers fluctuate with feeding activity due to diel rhythmicity of feeding behavior (Al Baki et al., 2018a). Especially, feeding, including nutrient uptake, can stimulate *Mv-ILP* expression and lead to a basal level of trehalose titer, while starvation can suppress *Mv-ILP* expression and lead to a hypertrehalosemic state (Al Baki et al., 2019a). Trehalose homeostasis is mainly controlled by enzyme activities of TPS for trehalose synthesis and TRE for trehalose degradation (Becker et al., 1996). Silencing *TPS* can significantly downregulate hemolymph trehalose level, while silencing *TRE* expression can upregulate its level in *M. vitrata* (Al Baki et al., 2019a). In our current study, ILP mutants showed upregulated *TPS* expression but downregulated *TRE* expression. Thus, the hypertrehalosemic state of these mutants might be due to the lack of ILP’s mediation effect on trehalose titer in the hemolymph of *M. vitrata*.

Two deletion mutants showed altered expression profiles of four IIS components and impaired immature development of *M. vitrata*. A previous study has identified seven IIS components in *M. vitrata*, including *Mv-InR*, *Mv-IRS*, *Mv-PI3K*, *Mv-PTEN* (phosphatase and tensin homolog), *Mv-FOXO*, *Mv-Akt*, and *Mv-TOR* (Al Baki et al., 2019b). Among these components, four components (*Mv-InR*, *Mv-Akt*, *Mv-FOXO*, and *Mv-TOR*) have been experimentally assessed. They are known to mediate hemolymph sugar level and immature development (Al Baki et al., 2018a, 2020). InR has been regarded as the sole ILP receptor for most insects (Wu and Brown, 2006; Wen et al., 2010). Although its expression fluctuates during immature development (Iga and Smagghe, 2011), its RNAi usually alters immature development. For example, expression levels of two *InR*s are increased during nymph-adult transition in *Aphis* (*Toxoptera*) *vitricidus* (brown citrus aphid), and their RNAi treatments result in a variety of malformed adult phenotypes along with significant nymphal mortality (Ding et al., 2017). In *Drosophila*, FOXO activation under little ILP signal can increase *InR* expression (Puig and Tjian, 2005). Thus, the increase of *Mv-InR* expression in ILP mutants of *M. vitrata* might be explained by the lack of ILPs and the increase of *Mv-FOXO* expression. Deletion mutants of *M. vitrata* showed increased expression of *Mv-Akt*. In *Drosophila*, Akt is activated by PDK or TOR to phosphorylate S6 ribosomal kinase (S6K), stimulating protein translation for cell growth (Sarbassov et al., 2005). Thus, in deletion mutants of *ILP*, Akt might not be activated because of the lack of ILP and the low expression level of *Mv-TOR*. Although FOXO is a transcriptional activator in the nucleus, its phosphorylation by Akt prevents its entry into the nucleus (Mattila et al., 2009). FOXO regulates body size by regulating cell numbers in *Drosophila* (Puig et al., 2003). Thus, *FOXO* RNAi can promote immature development in insects. Indeed, RNAi of *FOXO* expression in *Gryllus bimaculatus* (cricket) can increase its body size (Dabour et al., 2011). FOXO also regulates immature developmental rate in *M. vitrata* (Al Baki et al., 2018b). RNAi of *Mv-FOXO* expression led to developmental retardation. This is recapitulated in our current study, in which deletion mutants of *M. vitrata* exhibited a significant reduction in *Mv-FOXO* expression, leading to retardation of immature development. TOR pathway is activated by nutrient-sensing through amino acid transporters. It activates S6K and 4E-BP to initiate protein translation (Colombani et al., 2003). In *M. vitrata*, the TOR pathway is required for promoting larval development and adult reproduction (Al Baki et al., 2018a, 2019b). Especially, RNAi of *Mv-TOR* expression adversely influenced the larval developmental rate in *M. vitrata* (Al Baki et al., 2018b). Thus, the reduction of *Mv-TOR* expression in CRISPR mutants of *M. vitrata* explains the impairment of larval development.

Two ILP deletion mutants of *M. vitrata* showed impaired adult reproduction. The two ILPs of *M. vitrata* can mediate ovarian development of adult females of *M. vitrata* (Al Baki et al., 2019a). Oocyte development in the ovariole begins with active mitotic activity in the germarium, followed by vitellogenesis incorporating Vg into growing oocytes and finalized chorion formation outside mature oocytes. IIS plays a crucial role in mediating the oogenesis of *M. vitrata*, especially in previtellogenic development and Vg synthesis (Al Baki et al., 2020). Our current study showed that ILP mutants prevented BrdU incorporation in the germarium, suggesting decreased oocyte production from stem cells. In *Drosophila*, IIS regulates germline stem cell proliferation (Drummond-Barbosa and Spradling, 2001; Shim et al., 2013). Ovarian growth is arrested at the previtellogenic stage in mutants of *Drosophila* lacking IIS components (Tatar et al., 2001). The decrease of Vg expression in ILP mutants of *M. vitrata* suggests that ILP plays a crucial role in mediating Vg expression as a nutritional sensor. In *Drosophila*, the fat body can sense amino acids and send a nutritional signal called a fat body-derived signal (Géminard et al., 2009). In response to such fat body-derived signals, insulin-producing cells in the brain can produce ILPs to directly or indirectly activate Vg production (Hansen et al., 2014). Thus, the lack of nutrient signal in these deletion mutants might have prevented Vg expression. This finding was supported by the rescue of Vg expression in ILP mutants of *M. vitrata* after adding porcine insulin.

Males of ILP mutants are likely to show impaired reproductive activity. When wild-type control females mated with the mutant males, they showed a significant reduction in fecundity, and their eggs suffered low hatch rates, suggesting that mutant males of *Mv-ILP1* or *Mv-ILP2* were impaired in mating behavior or sperm fertility. In *Drosophila*, spermatogenesis begins with cell division of germline stem cells in the testis under ILP mediation because IIS mutant males show decreased numbers of germline cells, in which ILP promotes cell cycle in the G2/M phase (Ueishi et al., 2009). These findings suggest that males of *Mv-ILP1* and *Mv-ILP2* mutants show poor spermatogenesis. This needs to be further examined in a subsequent study.

In summary, two mutant lines lacking *Mv-ILP1* or *Mv-ILP2* showed altered hemolymph sugar levels and immature development. They also showed poor reproductive activities. These suggest their functional overlap by sharing a common IIS. However, any single ILP was not enough to mediate the physiological processes. This emphasizes the requirement of both ILPs in *M. vitrata*, probably by performing their mediation cooperatively or synergistically. This interaction between two ILPs needs to be clarified in a future study.

## Data Availability Statement

The raw data supporting the conclusions of this article will be made available by the authors, without undue reservation.

## Author Contributions

YK conceived and designed the study. MA and JJ performed the experiments and analyzed the data. MA drafted the manuscript. MA, JJ, and YK refined and approved the final manuscript. All authors contributed to the article and approved the submitted version.

## Conflict of Interest

The authors declare that the research was conducted in the absence of any commercial or financial relationships that could be construed as a potential conflict of interest.

## Footnotes

1 http://chopchop.cbu.uib.no

## Supplementary Material

Supplementary Figure 1 Influence of deletion mutation of two insulin-like peptides (Mv-ILP) genes on reduction in the number of previtellogenic oocytes of *M. vitrata* females: mutants of *Mv-ILP1* (“ΔILP1”) and *Mv-ILP2* (“ΔILP2”). Ovaries from 5-day-old mutant females were collected, and their ovarioles were separated. Newly dividing cells were specifically recognized by BrdU incorporation (green), while their nuclei were stained with DAPI (blue).

Supplementary Table 1 Primers used in this study for expression analysis.

## References

[B1] Al Baki M. A. Jung J. K. Kim Y. (2018a). Regulation of hemolymph trehalose titers by insulin signaling in the legume pod borer, *Maruca vitrata* (Lepidoptera: Crambidae). *Peptides* 106 28–36. 10.1016/j.peptides.2018.06.006 29935203

[B2] Al Baki M. A. Jung J. K. Maharjan R. Yi H. Ahn J. J. Gu X. (2018b). Application of insulin signaling to predict insect growth rate in *Maruca vitrata* (Lepidoptera: Crambidae). *PLoS One* 13:e0204935. 10.1371/journal.pone.0204935 30286156 PMC6171882

[B3] Al Baki M. A. Jung J. K. Kim Y. (2020). Alteration of insulin signaling to control insect pest by using transformed bacteria expressing dsRNA. *Pest Manag. Sci.* 76 1020–1030. 10.1002/ps.5612 31503391

[B4] Al Baki M. A. Lee D. W. Jung J. K. Kim Y. (2019a). Insulin-like peptides of the legume pod borer, *Maruca vitrata*, and their mediation effects on hemolymph trehalose level, larval development, and adult reproduction. *Arch. Insect Biochem. Physiol.* 100:e21524. 10.1002/arch.21524 30536703

[B5] Al Baki M. A. Lee D. W. Jung J. K. Kim Y. (2019b). Insulin signaling mediates previtellogenic development and enhances juvenile hormone-mediated vitellogenesis in a lepidopteran insect, *Maruca vitrata*. *BMC Dev. Biol.* 19:14. 10.1186/s12861-019-0194-8 31277577 PMC6610926

[B6] Badisco L. Van Wielendaele P. Vanden Broeck J. (2013). Eat to reproduce: a key role for the insulin signaling pathway in adult insects. *Front. Physiol.* 4:202. 10.3389/fphys.2013.00202 23966944 PMC3735985

[B7] Becker A. Schloder P. Steele J. E. Wegener G. (1996). The regulation of trehalose metabolism in insects. *Experientia* 52 433–439. 10.1007/bf01919312 8706810

[B8] Bustin S. A. Benes V. Garson J. A. Hellemans J. Huggett J. Kubista M. (2009). The MIQE guidelines: minimum information for publication of quantitative real-time PCR experiments. *Clin. Chem.* 55 611–622. 10.1373/clinchem.2008.112797 19246619

[B9] Chang J. C. Ramasamy S. (2014). Identification and expression analysis of diapause hormone and pheromone biosynthesis activating neuropeptide (DH-PBAN) in the legume pod borer, *Maruca vitrata* Fabricius. *PLoS One* 9:e84916. 10.1371/journal.pone.0084916 24409312 PMC3883689

[B10] Colombani J. Raisin S. Pantalacci S. Radimerski T. Montagne J. Léopold P. (2003). A nutrient sensor mechanism controls *Drosophila* growth. *Cell* 114 739–749. 10.1016/s0092-8674(03)00713-x 14505573

[B11] Dabour N. Bando T. Nakamura T. Miyawaki K. Mito T. Ohuchi H. (2011). Cricket body size is altered by systemic RNAi against insulin signaling components and epidermal growth factor receptor. *Dev. Growth Differ.* 53 857–869. 10.1111/j.1440-169x.2011.01291.x 21777227

[B12] Das D. Arur S. (2017). Conserved insulin signaling in the regulation of oocyte growth, development, and maturation. *Mol. Reprod. Dev.* 84 444–459. 10.1002/mrd.22806 28379636 PMC5477485

[B13] Ding B. Y. Shang F. Zhang Q. Xiong Y. Yang Q. Niu J. Z. (2017). Silencing of two insulin receptor genes disrupts nymph-adult transition of alate brown citrus aphid. *Int. J. Mol. Sci.* 18:357. 10.3390/ijms18020357 28230772 PMC5343892

[B14] Doudna J. A. Charpentier E. (2014). Genome editing. The new frontier of genome engineering with CRISPR-Cas9. *Science* 346:1258096. 25430774 10.1126/science.1258096

[B15] Drummond-Barbosa D. Spradling A. C. (2001). Stem cells and their progeny respond to nutritional changes during *Drosophila* Oogenesis. *Dev. Biol.* 231 265–278. 10.1006/dbio.2000.0135 11180967

[B16] Géminard C. Rullifson E. J. Leopold P. (2009). Remote control of insulin secretion by fat body cells in *Drosophila*. *Cell Metab.* 10 199–207. 10.1016/j.cmet.2009.08.002 19723496

[B17] Hansen I. A. Attardo G. M. Rodriguez S. D. Drake L. L. (2014). Four-way regulation of mosquito yolk protein precursor genes by juvenile hormone-, ecdysone-, nutrient-, and insulin-like peptide signaling pathways. *Front. Physiol.* 5:103. 10.3389/fphys.2014.00103 24688471 PMC3960487

[B18] Iga M. Smagghe G. (2011). Relationship between larval-pupal metamorphosis and transcript expression of insulin-like peptide and insulin receptor in *Spodoptera littoralis*. *Peptides* 32 531–538. 10.1016/j.peptides.2010.10.033 21056070

[B19] Jung J. K. Seo B. Y. Cho C. R. Kwon Y. H. Kim G. H. (2009). Occurrence of lepidopteran insect pests and injury aspects in Adzuki bean fields. *Korean J. Appl. Entomol.* 48 29–35. 10.5656/ksae.2009.48.1.029

[B20] Lenaerts C. Monjon E. Van Lommel J. Verbakel L. Vanden Broeck J. (2019). Peptides in insect oogenesis. *Curr. Opin. Insect Sci.* 31 58–64. 10.1016/j.cois.2018.08.007 31109674

[B21] Livak K. J. Schmittgen T. D. (2001). Analysis of relative gene expression data using real-time quantitative PCR and the 2^–Δ^ ^Δ^ ^CT^ Method. *Methods* 25 402–408. 10.1006/meth.2001.1262 11846609

[B22] Mattila J. Bremer A. Ahonen L. Kostiainen R. Puig O. (2009). *Drosophila* FOX regulates organism size and stress resistance through an adenylate cyclase. *Mol. Cell. Biol.* 29 5357–5365. 10.1128/mcb.00302-09 19651894 PMC2747984

[B23] Nagasawa H. Kataoka H. Isogai A. Tamura S. Suzuki A. Ishizaki H. (1984). Amino-terminal amino acid sequence of the silkworm prothoracicotropic hormone: homology with insulin. *Science* 226 1344–1345. 10.1126/science.226.4680.1344 17832633

[B24] Nässel D. R. Vanden Broeck J. (2016). Insulin/IGF signaling in Drosophila and other insects: factors that regulate production, release and post-release action of the insulin-like peptides. *Cell. Mol. Life Sci.* 73 271–290. 10.1007/s00018-015-2063-3 26472340 PMC11108470

[B25] Nijhout H. F. Callier V. (2013). A new mathematical approach for qualitative modeling of the insulin-TOR-MAPK network. *Front. Physiol.* 4:245. 10.3389/fphys.2013.00245 24062690 PMC3771213

[B26] Okamoto N. Yamanaka N. (2015). Nutrition-dependent control of insect development by insulin-like peptides. *Curr. Opin. Insect Sci.* 11 21–30. 10.1016/j.cois.2015.08.001 26664828 PMC4671074

[B27] Park Y. Kim Y. (2013). RNA interference of glycerol biosynthesis suppresses rapid cold hardening of the beet armyworm, *Spodoptera exigua*. *J. Exp. Biol.* 216 4196–4203. 23948473 10.1242/jeb.092031

[B28] Puig O. Marr M. T. Ruhf M. L. Tjian R. (2003). Control of cell number by *Drosophila* FOXO: downstream and feedback regulation of the insulin receptor pathway. *Genes Dev.* 17 2006–2020. 10.1101/gad.1098703 12893776 PMC196255

[B29] Puig O. Tjian R. (2005). Transcriptional feedback control of insulin receptor by dFOXO/FOXO1. *Genes Dev.* 19 2435–2446. 10.1101/gad.1340505 16230533 PMC1257398

[B30] Rajan A. Perrimon N. (2012). *Drosophila* cytokine unpaired 2 regulates physiological homeostasis by remotely controlling insulin secretion. *Cell* 151 123–137. 10.1016/j.cell.2012.08.019 23021220 PMC3475207

[B31] Rullifson E. J. Kim S. K. Nusse R. (2002). Ablation of insulin-producing neurons in flies: growth and diabetic phenotypes. *Science* 296 1118–1120. 10.1126/science.1070058 12004130

[B32] Sano H. Nakamura A. Texada M. J. Truman J. W. Ishimoto H. Kamikouchi A. (2015). The nutrient-responsive hormone CCHamide-2 controls growth by regulating insulin-like peptides in the brain of *Drosophila melanogaster*. *PLoS Genet.* 11:e1105481. 10.1371/journal.pgen.1005481 26020940 PMC4447355

[B33] Sarbassov D. D. Guertin D. A. Ali S. M. Sabatini D. M. (2005). Phosphorylation and regulation of Akt/PKB by the rictor-mTOR complex. *Science* 307 1098–1101. 10.1126/science.1106148 15718470

[B34] SAS Institute, (1989). *SAS/STAT User’s Guide.* Cary, NC: SAS Institute, Inc.

[B35] Sharma H. C. (1998). Bionomics, host plant resistance, and management of the legume pod borer *Maruca vitrata* - a review. *Crop Prot.* 17 373–386. 10.1016/s0261-2194(98)00045-3

[B36] Shim J. Gururaja-Rao S. Banerjee U. (2013). Nutritional regulation of stem and progenitor cells in *Drosophila*. *Development* 140 4647–4656. 10.1242/dev.079087 24255094 PMC3833425

[B37] Sun D. Guo Z. Liu Y. Zhang Y. (2017). Progress and prospects of CRISPR/Cas systems in insects and other arthropods. *Front. Physiol.* 8:608. 10.3389/fphys.2017.00608 28932198 PMC5592444

[B38] Tatar M. Kopelman A. Epstein D. Tu M. P. Yin C. M. Garofalo R. S. (2001). A mutant *Drosophila* insulin receptor homolog that extends life-span and impairs neuroendocrine function. *Science* 292 107–110. 10.1126/science.1057987 11292875

[B39] Ueishi S. Shimizu H. H. Inoue Y. (2009). Male germline stem cell division and spermatocyte growth require insulin signaling in *Drosophila*. *Cell Struct. Funct.* 34 61–69. 10.1247/csf.08042 19384053

[B40] Veenstra J. A. (2020). Arthropod IGF, relaxin and gonadulin, putative orthologs of *Drosophila* insulin-like peptides 6, 7 and 8, likely originated from an ancient gene triplication. *PeerJ* 8:e9534. 10.7717/peerj.9534 32728497 PMC7357564

[B41] Wen Z. Gulia M. Clark K. D. Dhara A. Crim J. W. (2010). Two insulin-like peptide family members from the mosquito *Aedes aegypti* exhibit differential biological and receptor binding activities. *Mol. Cell. Endocrinol.* 328 47–55. 10.1016/j.mce.2010.07.003 20643184 PMC2957182

[B42] Wu Q. Brown M. R. (2006). Signaling and function of insulin-like peptides in insects. *Annu. Rev. Entomol.* 51 1–24. 10.1146/annurev.ento.51.110104.151011 16332201

