# Supplementary material for: Physiological Alterations in Deletion Mutants of Two Insulin-Like Peptides Encoded in Maruca vitrata Using CRISPR/Cas9
Source: Front Physiol. 2021 Jul 2;12:701616. doi: 10.3389/fphys.2021.701616 (PMC8284963; doi:10.3389/fphys.2021.701616)
Supplement: Supplementary Figure 1 — Influence of deletion mutation of two insulin-like peptides (Mv-ILP) genes on reduction in the number of previtellogenic oocytes of M. vitrata females: mutants of Mv-ILP1 (“ΔILP1”) and Mv-ILP2 (“ΔILP2”). Ovaries from 5-day-old mutant females were collected, and their ovarioles were separated. Newly dividing cells were specifically recognized by BrdU incorporation (green), while their nuclei were stained with DAPI (blue). [file Data_Sheet_1.doc]

**Table S1. Primers used in this study for expression analysis**

| **Genes** | **Primer sequence (5’ -3’)** | **Annealing temperature (°C)** | **Purposes** |
| --- | --- | --- | --- |
| **Mv-ILP1** | F: GAGATGATGAGCAAGGTCTG  R: GTGGTCCTCTAACTCGTCTA | 54 RT-PCR  RT-qPCR | |
| **Mv-ILP2** | F: GGCACGAGGACGTACAAGAT  R: TCAGGGCGTTCTTTGAGGAG | 58 | RT-PCR  RT-qPCR |
| **β-Actin** | F: CATCACCATCGGAAACGAAAGG  R: ATACTGTGTTGGCGTACAGGTC | 52 | RT-PCR  RT-qPCR |

**FIGURE S1.** Influence of deletion mutation of two insulin-like peptide (Mv-ILP) genes on reduction in the number of previtellogenic oocytes of *M. vitrata* females: mutants of *Mv-ILP1* (‘ΔILP1’) and *Mv-ILP2* (‘ΔILP2’). Ovaries from 5 days old mutant female were collected and their ovarioles were separated. Newly dividing cells were specifically recognized by BrdU incorporation (green) while their nuclei were stained with DAPI (blue).

**
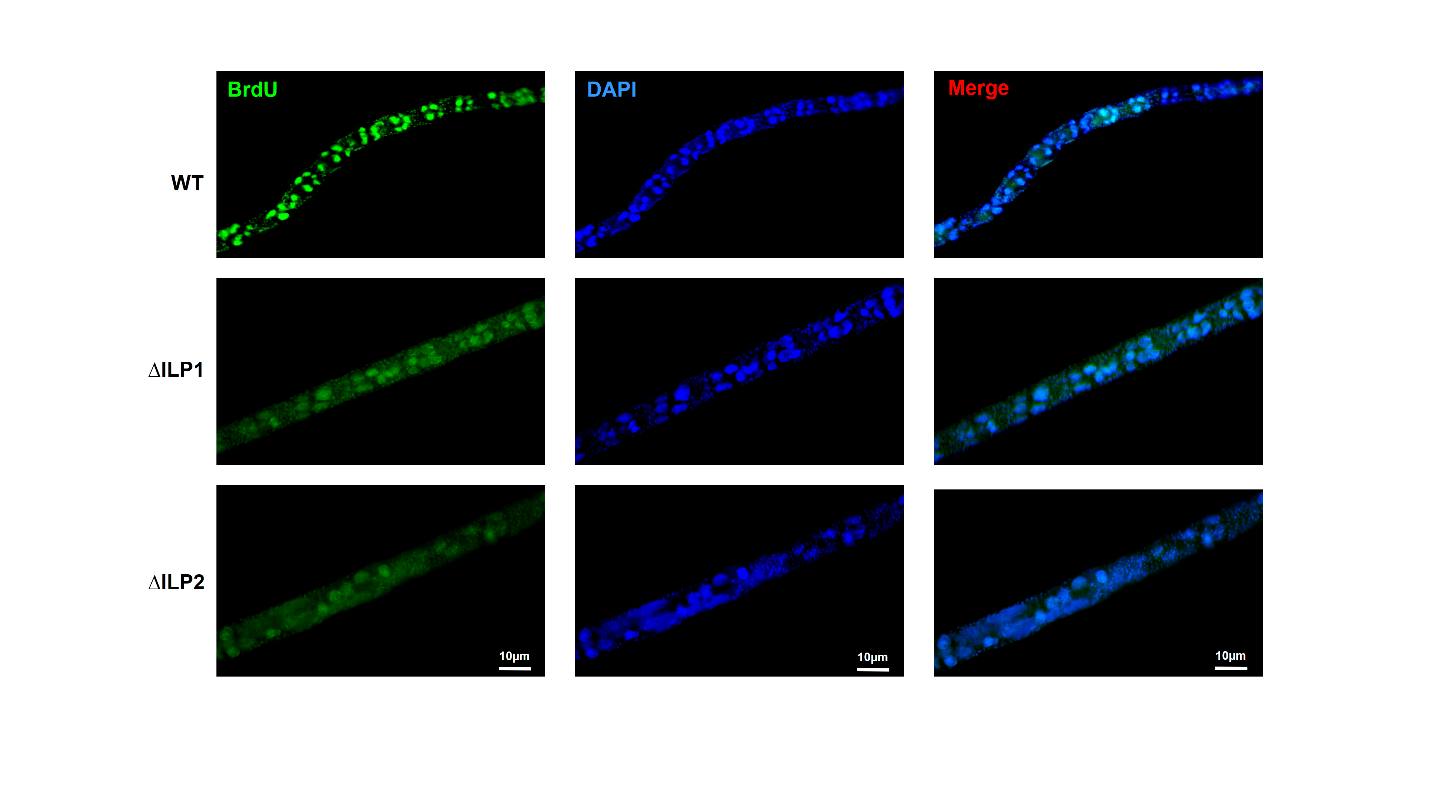
**
